# Hospital Administration

**Published:** 1893-10-07

**Authors:** A. C. Davis

**Affiliations:** Secretary, Chelsea Hospital for Women


### HOSPITAL ADMINISTRATION.

Referring to the article in your issue of the 23rd ult. in which we are charged with a heavy percentage of outlay for administration as compared with maintenance, allow me to remind you that a high percentage of administration to maintenance may be arrived at where the expenses of management are but normal, and the maintenance expenditure admirably low, and surely economy in maintenance is as worthy of praise as extravagance in administration is deserving of censure. With regard to the details of our administration expenses, which you wish explained, I beg to point to a large item, "interest on loan." This correctly speaking is an interest on a mortgage debt, for which contingency I observe one of the hospitals you hold up as a pattern of good management, has a separate account. Another item to which I would call your attention is under the heading of special appeals; this sum, though not high, adds, again, to our percentage as compared with some of the institutions whose figures you give, and who, apparently, had no need to debit themselves one penny in respect of this usual and justifiable expenditure. The exclusion from our administration account of these two items alone would reduce our percentage to 16·247 as compared to maintenance; and to 13·150 as to total ordinary expenditure, a ratio which is actually lower than that for which you have bestowed congratulations upon a similar hospital. In conclusion I trust I may be pardoned for suggesting that the following facts should not be lost sight of in making calculations upon which to found a basis for a decision regarding the excellence or otherwise of the management of an institution. (*A*) That a low percentage of administration to maintenance may so work out, not of necessity because the management is conducted upon the most approved principles of economy, but, possibly, from the fact of maintenance being extravagantly high. (*B*) That institutions having no immediate need of funds are able to reduce their expenditure upon administration through their ability to dispense with the costly process of appealing, to which poorer charities are compelled to resort.—Yours faithfully. A. C. Davis.

Secretary, Chelsea Hospital for Women.

